# Stretch regulated surface roughness controls macrophage inflammatory polarization through adhesion–chromatin coupling

**DOI:** 10.3389/fphar.2026.1857290

**Published:** 2026-05-28

**Authors:** Gang Yang, Yi Han, Tao Tang, Xu Liu, Hong-yu He, Yong-fu Xiong, Jun-wei Wang, Xiao-song Qing, Chen Zhang

**Affiliations:** 1 Department of General Surgery, Affiliated Hospital of North Sichuan Medical College, National Clinical Key Specialty (General Surgery), Sichuan Branch of National Clinical Research Center for Digestive Diseases, Sichuan Clinical Research Center for Digestive Diseases, Nanchong, China; 2 Anesthesia and Surgery Center of the Affiliated Hospital of North Sichuan Medical College, Nanchong, China; 3 Department of General Surgery, Langzhong Hospital Affiliated to North Sichuan Medical College, Langzhong, China; 4 Department of General Surgery, Zigong Fourth People’s Hospital, Zigong, Sichuan, China

**Keywords:** chromatin accessibility, focal adhesion kinase, immunomodulatory biomaterials, macrophage polarization, PDMS, surface roughness

## Abstract

**Introduction:**

Macrophages are early responders to biomaterial interfaces, but the specific role of surface roughness in regulating macrophage inflammatory phenotypes remains difficult to distinguish from the effects of substrate stiffness and other material properties. This study aimed to isolate the effect of surface roughness and clarify its role in macrophage–material interactions and inflammatory programming.

**Methods:**

We developed a magnetic stretching device to impose controlled deformation on a thin polydimethylsiloxane (PDMS) membrane, thereby tuning its surface roughness. Atomic force microscopy was used to characterize surface topography and Young’s modulus. RAW264.7 macrophages were cultured on stretched low-roughness membranes to evaluate cell morphology, cytoskeletal organization, focal adhesion kinase signaling, chromatin features, inflammatory marker expression, cytokine secretion, phagocytic activity, and adhesion force. NaOH-treated glass was further used as an independent material platform to validate the effects of roughness modulation.

**Results:**

Stretching significantly reduced PDMS surface roughness without measurably altering Young’s modulus. Macrophages cultured on stretched low-roughness membranes showed increased spreading, reduced roundness, decreased F-actin intensity, and enhanced focal adhesion kinase signaling, suggesting altered cytoskeletal organization and stronger cell–material interactions. These changes were accompanied by increased chromatin density and decreased chromatin accessibility, while nuclear area remained unchanged. Functionally, stretched membranes promoted a pro-inflammatory phenotype, as indicated by increased TNF and IL-1 expression, elevated TNF-α secretion, enhanced phagocytic activity, and increased adhesion force measured by single-cell force spectroscopy. Validation experiments on NaOH-treated glass further confirmed that roughness modulation affected macrophage adhesion, chromatin accessibility, and inflammatory output across material platforms.

**Discussion:**

These findings identify surface roughness as a mechanically tunable regulator of macrophage inflammatory polarization. The results further suggest that roughness-dependent changes in cell–material adhesion may influence chromatin organization and accessibility, providing a potential adhesion-associated chromatin mechanism underlying macrophage inflammatory programming at biomaterial interfaces.

## 1 Introduction

Macrophages are central regulators of inflammation, tissue repair, and host responses to biomaterial interfaces (Russell et al., 2019). Their phenotypic plasticity enables them to adapt rapidly to environmental cues and adopt functionally distinct inflammatory states, broadly simplified as pro-inflammatory and anti-inflammatory programs (Liddiard and Taylor, 2015). Because macrophages are among the first cells to sense implanted or engineered materials, understanding how material-associated cues direct macrophage behavior is of major importance for pharmacology, regenerative medicine, and immunomodulatory biomaterial design (Brown et al., 2014). In particular, controlling macrophage activation at material interfaces has emerged as a promising strategy to improve tissue integration, modulate local inflammatory responses, and enhance therapeutic outcomes.

In addition to soluble biochemical signals, macrophages are highly sensitive to the physical properties of their microenvironment. Accumulating studies have shown that stiffness, topography, ligand presentation, and mechanical stress can each influence macrophage morphology, adhesion, cytoskeletal organization, and downstream inflammatory phenotype (Ni et al., 2023; Clevenger et al., 2025). These effects are commonly mediated through mechanotransductive pathways involving focal adhesions, actomyosin remodeling, and nucleus-associated chromatin regulation (Su and Yin, 2025; Jain et al., 2022). However, in many engineered systems, multiple physical properties change simultaneously, making it difficult to isolate the specific contribution of a single cue. As a result, the role of surface roughness in macrophage inflammatory regulation remains incompletely understood, particularly when decoupled from changes in bulk mechanical properties.

Surface roughness is a key interfacial feature that directly affects cell attachment, membrane contact, force transmission, and protein adsorption (Li et al., 2023). Previous studies have suggested that roughness can regulate immune cell behavior, yet these observations are often confounded by parallel changes in substrate stiffness, chemistry, wettability, or fabrication-induced heterogeneity (Dabare et al., 2023). For macrophages, such coupling effects are especially problematic because adhesion-dependent signaling strongly influences their polarization state (Xiong et al., 2025). Thus, a system that selectively tunes surface roughness while preserving bulk modulus would provide an effective platform for dissecting how topographical changes alone reshape macrophage mechanosensing and inflammatory fate.

Mechanical stretch provides a potentially useful but underexplored approach to regulate surface structure in elastomeric membranes. Polydimethylsiloxane (PDMS) is widely used in mechanobiology because of its biocompatibility, optical transparency, and tunable mechanical properties. Beyond acting as a deformable substrate, PDMS may also undergo stretch-associated changes in surface morphology that alter the physical interface experienced by adherent cells. Whether such stretch-induced topographical changes are sufficient to instruct macrophage behavior independently of bulk stiffness remains unclear. Moreover, how these interfacial changes are coupled with cytoskeletal remodeling, adhesion force, and chromatin organization in macrophages has not been systematically investigated.

Emerging evidence indicates that macrophage phenotypes are not only determined by membrane receptors and signaling cascades, but also by biophysical regulation of nuclear state and chromatin organization (Lin et al., 2022). Changes in cell spreading and adhesion can propagate through the cytoskeleton to the nucleus, influencing nuclear mechanics, chromatin compaction, and transcriptional accessibility (Zhang et al., 2025). This adhesion–cytoskeleton–chromatin axis provides a plausible mechanistic framework linking surface topography to inflammatory programming. However, direct evidence connecting roughness-dependent adhesion strength with chromatin remodeling and macrophage polarization is still limited.

In this study, we developed a mechanical device that imposes stretch on a thin PDMS membrane to generate a low-roughness surface state without significantly altering substrate modulus. Using atomic force microscopy, we found that stretching reduced PDMS surface roughness while preserving bulk mechanical stiffness. Macrophages cultured on stretched membranes exhibited altered morphology, reduced F-actin organization and roundness, increased cell area and focal adhesion kinase expression, and enhanced adhesion force. These changes were accompanied by increased chromatin condensation and a shift toward a pro-inflammatory phenotype. To further validate the role of surface roughness, we independently increased glass roughness by NaOH treatment and observed an opposite macrophage response. Together, these findings support a model in which surface roughness regulates macrophage inflammatory polarization through adhesion-associated control of cytoskeletal and chromatin states, and identify interfacial topography as a mechanically tunable parameter for the design of immunomodulatory biomaterials.

## 2 Materials and methods

### 2.1 Pre-stretch device fabrication

The Polydimethylsiloxane (PDMS) membranes were prepared from a elastomer kit (Sylgard 184, Dow Corning) according to the manufacturer’s instructions. The base and curing agent were mixed at a 10:1 (w/w) ratio, degassed under vacuum, cast onto a flat mold, and cured at 60 °C for 12 h to obtain thin membranes. After curing, the PDMS sheets were cut into rectangular strips of defined dimensions and mounted on a custom-built uniaxial pre-stretch device. The device was fabricated by 3D printing and consisted of two opposing clamps that secured both ends of the membrane and allowed controlled uniaxial elongation along its long axis. Stretch was applied by increasing the distance between the clamps, thereby imposing tensile preconditioning on the membrane before cell seeding.

### 2.2 Cell culture

The macrophage cell line RAW264.7 cells were obtained from the American Type Culture Collection (ATCC, Manassas, VA, United States) and cultured in Advanced Dulbecco’s Modified Eagle’s Medium (DMEM; Invitrogen) supplemented with 10% fetal bovine serum (FBS; Fisher Scientific, Houston, TX, United States) and 1% (v/v) penicillin–streptomycin. Prior to cell seeding, PDMS membranes were coated with poly-amino acid (10 mg/mL). Cells were seeded onto the coated membranes and used at approximately 80% confluence. Cultures were maintained at 37 °C in a humidified atmosphere containing 5% CO_2_. Cells were washed with phosphate-buffered saline (PBS; Corning, 46-013-CM) and passaged by gentle medium flushing. To minimize potential differences in adhesion ligand density, PDMS stretching or NaOH treatment was performed before surface coating. All substrates were then coated using the same poly-amino-acid reagent, concentration, incubation time, and temperature before cell seeding. This workflow was designed to ensure comparable coating conditions across groups.

### 2.3 Atomic force microscopy characterization of PDMS membranes

Surface topography and local mechanical properties of PDMS membranes were characterized by atomic force microscopy (AFM; NanoWizard V, Bruker) using a Bruker SNL-10 cantilever. For topographical characterization, membranes from the 0% and 20% stretch groups were scanned in PeakForce Tapping mode to obtain surface height maps. Step height and surface roughness were quantified from the AFM images using the manufacturer’s analysis software. For mechanical characterization, AFM-based force spectroscopy was performed with the same cantilever. Force–distance curves were acquired at multiple locations on each membrane, and the local Young’s modulus was calculated by fitting the approach curves with the Sneddon model. Multiple regions were analysed for each sample, and the averaged values were used for statistical analysis. Single-cell force spectroscopy (SCFS) was performed using individual RAW264.7 cells attached to a ConA-functionalized tipless cantilever (Bruker NPO-10). To achieve stable cell capture, cantilevers were sequentially coated with biotin-BSA, streptavidin, and biotin-ConA, enabling strong binding to cell-surface glycoconjugates. For cell attachment, the cantilever was gently brought into contact with a single cell using a loading force of 15 nN for 10 s, followed by a 10 min stabilization period to allow firm adhesion and minimize detachment during subsequent measurements. Successful cell attachment was confirmed under an inverted microscope, and cells showing lateral displacement were excluded. The cell-bearing cantilever was then used to probe cell–substrate detachment in HEPES buffer under identical loading conditions. Both the approach and retraction velocities were set to 3 μm/s to minimize hydrodynamic drag and ensure reproducible force–distance measurements. All SCFS experiments were performed under temperature-controlled conditions at 37 °C, and adhesion forces were extracted from the force–distance curves using Nanoscope Analysis software.

### 2.4 Immunofluorescence staining

Cells were fixed in pre-chilled 4% paraformaldehyde (PFA) for 15 min at room temperature and permeabilized with 0.5% Triton X-100 (Sigma, T8787) in PBS for 10 min. Samples were then blocked with 1% normal donkey serum and gamma globulins (NDS) in PBS for 1 h at room temperature. Cells were incubated with the primary antibody against FAK (Santa Cruz Biotechnology, United States; 1:200) for 2 h at room temperature, followed by incubation with goat anti-rabbit IgG (H + L) cross-adsorbed secondary antibody (1:400) and Alexa Fluor 488-conjugated phalloidin (Thermo Fisher; 1:800) for 1 h at room temperature. Nuclei were counterstained with 4′,6-diamidino-2-phenylindole (DAPI; Invitrogen Molecular Probes, D3571) for 5 min according to the manufacturer’s instructions. Between each step, samples were washed three times with PBS for 5 min to remove unbound reagents. Fluorescence images were acquired using a Zeiss fluorescence microscope. Cell morphology was quantified using ImageJ, with roundness used as a morphological parameter. For each condition, at least 30 cells from three independent experiments were analysed.

### 2.5 Macrophage phagocytosis assay

Macrophage phagocytic function was determined using pHrodo™ Red BioParticles (Thermo Fisher Scientific, P35361), a pH-sensitive probe that becomes fluorescent after particle uptake into acidic intracellular compartments. RAW264.7 cells were plated in 96-well plates at 2 × 104 cells per well and allowed to attach for at least 1 h before treatment. Following the indicated treatment for 2 h, pHrodo BioParticles were prepared in PBS at 1 mg/mL, mixed thoroughly, diluted 1:10 in complete medium, and added to the cultures. After a further 2 h incubation at 37 °C, cells were rinsed with PBS to remove extracellular particles and fixed in 4% paraformaldehyde for 10 min at room temperature. Nuclear staining was then performed with DAPI (1:1,000) for 5 min. Images were captured using a fluorescence microscope with identical acquisition parameters for all groups. Phagocytic activity was quantified by randomly analysing at least 200 cells per group and calculating the proportion of cells showing intracellular red fluorescence.

### 2.6 Quantitative real-time PCR

Total RNA was extracted from RAW264.7 cells cultured on different PDMS membranes using RNA extraction kit (ThermoFisher Biotechnology, United States). RNA concentration and purity were determined by nano-drop (ThermoFisher Biotechnology, United States), and equal amounts of RNA were reverse-transcribed into cDNA using reverse transcription kit (Bio-rad). Quantitative real-time PCR (qRT-PCR) was performed using SYBR Green or probe-based master mix (Bio-rad), on a PCR instrument. The expression of macrophage polarization-related genes, including TNFα, IL-1β, Arg1, and IL-10, was measured using gene-specific primers. Relative mRNA expression levels were calculated using the 2^-ΔΔCt^ method with GAPDH as the internal reference. The primers used in the study including: TNF F: CCC​TCA​CAC​TCA​GAT​CAT​CTT​CT, R: GCTACGACGTGGG CTACAG; IL-1 F:GCAACTGTTCCTGAACTCAACT, R: ATC​TTT​TGG​GGT​CCG​TCA​ACT; Arg1 F:CTGGGGATTGGCAAGGTGAT, R: CAG​CCC​GTC​GAC​ATC​AAA​G; IL-10 F: GTA GAAGTGATGCCCCAGGC, R: GAC​ACC​TTG​GTC​TTG​GAG​CTT​ATT.

### 2.7 Enzyme linked immunosorbent assay (ELISA)

The secretion of TNF-α, IL-1β and IL-10 was measured in culture supernatants using commercial ELISA kits (Abclonal, RK00016, rk04878 and RK03242) according to the manufacturer’s instructions. After macrophages were cultured on the different PDMS membranes for 48 h, supernatants were collected, centrifuged to remove debris, and subjected to ELISA. Absorbance was read at 570 nm using a microplate reader (Bio-Rad), and cytokine concentrations were calculated from standard curves.

### 2.8 Data statistic

All experiments were performed with at least three independent biological replicates. Data are presented as mean ± SD. Statistical comparisons between two groups were performed using an unpaired two-tailed Student’s t-test, while comparisons among multiple groups were performed using one-way ANOVA followed by appropriate *post hoc* tests. A value of P < 0.05 was considered statistically significant.

## 3 Results

### 3.1 Construction of a magnetic stretching device and AFM characterization of stretch-regulated PDMS surface properties

**FIGURE 1:**
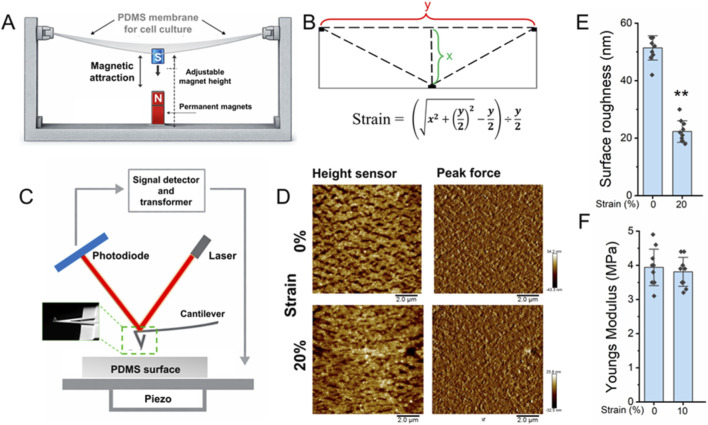
Design of the magnetic stretching device and AFM characterization of stretch-regulated PDMS surface properties. **(A)** Schematic illustration of the custom-built magnetic device used to impose controlled deformation on a thin PDMS membrane for cell culture. The membrane was fixed at both ends, and magnetic attraction generated a central downward displacement. **(B)** Schematic showing the geometric parameters used to calculate membrane strain, where y represents the initial span between the two fixed ends and x represents the central vertical displacement after deformation. **(C)** Schematic of the atomic force microscopy (AFM) setup used for surface topography and mechanical characterization of PDMS membranes. A laser reflected from the cantilever onto a photodiode detector was used to monitor cantilever deflection during scanning. **(D)** Representative AFM height sensor and PeakForce images of PDMS membranes in the non-stretched (0%) and stretched (20%) groups. Scale bars, 2.0 μm. **(E)** Quantification of PDMS surface roughness in the 0% and 20% stretch groups. **(F)** Quantification of Young’s modulus of PDMS membranes in the indicated groups. Data are presented as mean ± SD. **p < 0.01.

To investigate how mechanically induced surface changes regulate macrophage behaviour, we first developed a magnetic device capable of imposing controllable stretch on a thin PDMS membrane used for cell culture (Figure 1A). In this system, the membrane was fixed at both ends, while a magnetic attraction module positioned beneath the central region generated a downward displacement of the membrane. By adjusting the height of the upper magnet relative to the permanent magnet below, the membrane could be deflected to different extents, thereby generating tensile strain across the culture surface without direct contact with the cell-seeded region.

The strain applied to the membrane was quantified based on the geometric change in membrane length after deformation (Figure 1B). Specifically, the initial horizontal distance between the two fixed ends was defined as y, and the central downward deflection was defined as x. After magnetic loading, the membrane adopted two symmetric slanted segments, and the deformed membrane length was calculated from the sum of the two segment lengths. Engineering strain was then determined as the relative increase in membrane length compared with the initial undeformed length, according to:

ε=x2+y22−y2y2

where εis the applied strain, y is the initial membrane span, and xis the central vertical displacement. Based on this relationship, the deformation condition used for subsequent experiments corresponded to the indicated strain levels. To determine whether stretching altered the physical interface sensed by cells, we next examined the membrane surface using atomic force microscopy (AFM). A schematic of the AFM setup is shown in Figure 1C. Briefly, a laser beam was reflected from the cantilever onto a photodiode detector, and surface topography was reconstructed from cantilever deflection during probe scanning across the PDMS membrane.

AFM imaging revealed a clear difference in surface morphology between non-stretched and stretched PDMS membranes (Figure 1D). The non-stretched membrane displayed a relatively coarse and undulating topography, whereas the stretched membrane exhibited a visibly smoother and more uniform surface. Quantitative analysis confirmed that stretching significantly reduced the surface roughness of the PDMS membrane. In particular, the roughness decreased from approximately 50 nm in the non-stretched group to about 20 nm after 20% strain (Figure 1E), indicating that membrane stretching effectively rewrote the microscale interfacial topography. Because surface topography and substrate mechanics often change together, we next asked whether membrane stretching also altered the bulk stiffness of PDMS. AFM-based mechanical measurements showed that the Young’s modulus remained largely unchanged after stretching, with no significant difference between the control and stretched groups (Figure 1F). These data indicate that the applied stretch primarily modified surface roughness while preserving the bulk mechanical stiffness of the membrane.

Together, these results demonstrate that our magnetic stretching device provides a reliable method to mechanically tune the surface state of PDMS membranes. Importantly, stretching markedly reduced surface roughness without significantly affecting Young’s modulus, thereby establishing an experimental platform that largely decouples interfacial topography from bulk substrate stiffness for subsequent studies of macrophage mechanosensing and inflammatory polarization.

### 3.2 Stretched membranes alter macrophage morphology and adhesion associated cytoskeletal organization

**FIGURE 2:**
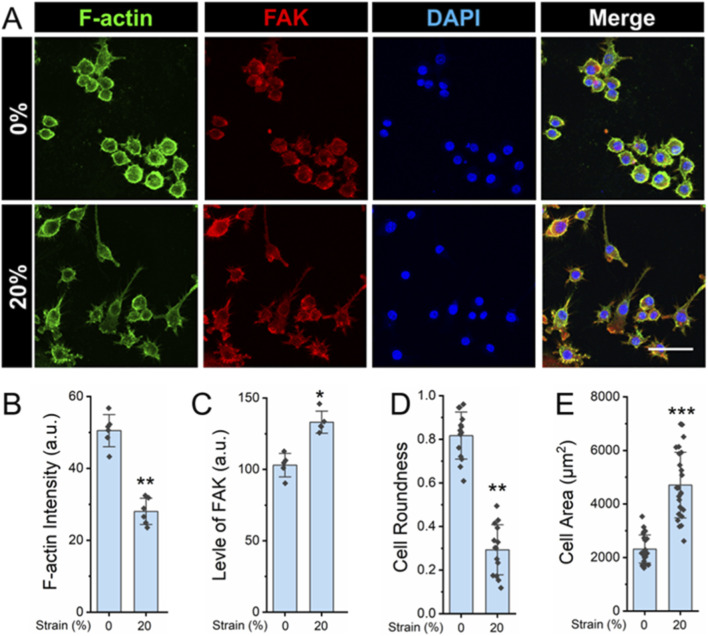
Stretched low-roughness membranes alter RAW264.7 macrophage morphology and adhesion-associated cytoskeletal organization. **(A)** Representative immunofluorescence images of RAW264.7 macrophages cultured on non-stretched (0%) and stretched (20%) PDMS membranes for 12 h, followed by staining for F-actin (green), focal adhesion kinase (FAK; red), and nuclei (DAPI; blue). Merged images are shown in the right panel. Scale bar, 50 μm. **(B)** Quantification of F-actin fluorescence intensity in RAW264.7 macrophages cultured on PDMS membranes with the indicated strain levels. **(C)** Quantification of FAK fluorescence intensity in RAW264.7 macrophages cultured on PDMS membranes with the indicated strain levels. **(D)** Quantification of cell roundness in macrophages cultured on non-stretched and stretched membranes. **(E)** Quantification of cell spreading area in macrophages cultured on non-stretched and stretched membranes. Data are presented as mean ± SD. Each dot represents one cell or one independent measurement. *p < 0.05, **p < 0.01, ***p < 0.001 versus the 0% group.

To determine how stretch-regulated surface changes influence macrophage behaviors, we cultured RAW264.7 macrophages on non-stretched and stretched PDMS membranes for 12 h, followed by immunofluorescence staining of F-actin, focal adhesion kinase (FAK), and nuclei (DAPI) (Figure 2A). This analysis was performed to assess whether the low-roughness surface generated by membrane stretching could alter macrophage cytoskeletal organization, adhesion-related signalling, and cell morphology during early cell–material interactions.

Immunofluorescence imaging revealed marked differences in cell morphology between the two groups. RAW264.7 cells cultured on the non-stretched membrane maintained a relatively rounded morphology with compact actin organization, whereas cells on the stretched membrane displayed a more spread and elongated appearance, with prominent protrusions and a broader attachment area (Figure 2A). These observations suggested that the stretched low-roughness surface promoted stronger cell–substrate interaction and altered macrophage structural organization.

Quantitative analysis further showed that RAW264.7 cells on the stretched membrane exhibited a marked reduction in F-actin intensity, with an approximately 0.56-fold level relative to the non-stretched group (Figure 2B). In contrast, FAK intensity increased to approximately 1.3-fold of the non-stretched control (Figure 2C). These data indicate that membrane stretching not only altered cell morphology, but also reshaped the balance between cytoskeletal organization and adhesion-associated signaling in macrophages. Consistent with these staining results, morphological quantification demonstrated that stretched membranes significantly reduced cell roundness to approximately 0.37-fold of the non-stretched group (Figure 2D), while cell area increased to approximately 2.0-fold of the control level (Figure 2E). Thus, RAW264.7 macrophages on the stretched membrane adopted a less circular and more extensively spread morphology, consistent with enhanced substrate engagement.

Together, these findings indicate that the stretch-treated PDMS membrane, which exhibits lower surface roughness, promotes RAW264.7 macrophage spreading and focal adhesion-associated signalling while reducing F-actin intensity and cell roundness. These early changes in cytoskeletal and morphological state suggest that stretch-regulated interfacial topography is sufficient to reprogram macrophage physical interaction with the substrate, potentially contributing to subsequent nuclear remodeling and inflammatory polarization.

### 3.3 Stretched membranes increase chromatin density and promote pro-inflammatory polarization of macrophages

**FIGURE 3:**
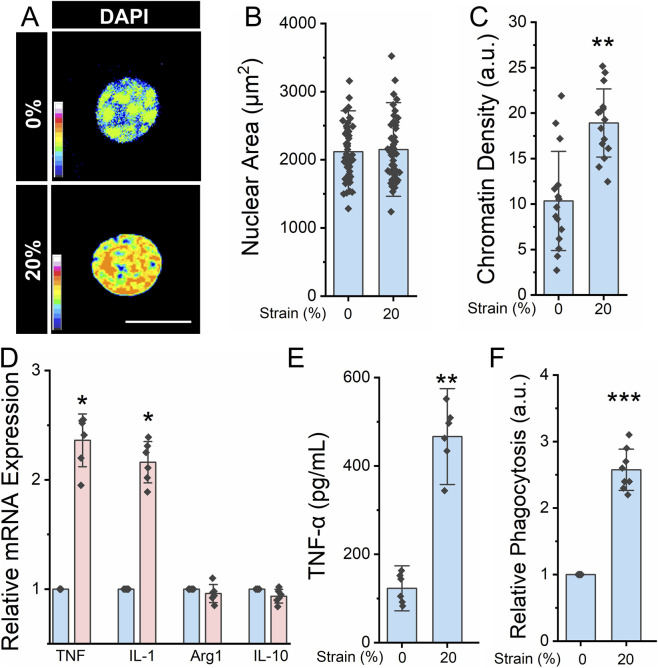
Stretched low-roughness membranes increase chromatin density and promote pro-inflammatory activation of RAW264.7 macrophages. **(A)** Representative DAPI images showing nuclear chromatin distribution in RAW264.7 macrophages cultured on non-stretched (0%) and stretched (20%) PDMS membranes for 12 h. Scale bar, 10 μm. **(B)** Quantification of nuclear area in RAW264.7 macrophages cultured on membranes with the indicated strain levels. **(C)** Quantification of chromatin density in RAW264.7 macrophages cultured on non-stretched and stretched membranes. **(D)** Relative mRNA expression of pro-inflammatory markers (TNF and IL-1) and anti-inflammatory markers (Arg1 and IL-10) in RAW264.7 macrophages cultured on PDMS membranes with the indicated strain levels, as determined by qRT-PCR. **(E)** Quantification of TNF-α secretion in the culture supernatant of RAW264.7 macrophages cultured on non-stretched and stretched membranes, as measured by ELISA. **(F)** Quantification of phagocytic activity in RAW264.7 macrophages cultured on PDMS membranes with the indicated strain levels. Data are presented as mean ± SD. Each dot represents one cell or one independent measurement. *p < 0.05, **p < 0.01, ***p < 0.001 versus the 0% group.

To further determine whether the stretch-regulated surface could affect nuclear organization and inflammatory phenotype, RAW264.7 macrophages were cultured on non-stretched and stretched PDMS membranes for 12 h, followed by analysis of nuclear morphology, chromatin density, inflammatory gene expression, cytokine secretion, and phagocytic function (Figure 3). Since cell adhesion and cytoskeletal remodeling can propagate mechanical signals to the nucleus, we first examined whether the low-roughness surface generated by membrane stretching altered nuclear architecture.

DAPI imaging revealed that RAW264.7 macrophages cultured on stretched membranes exhibited a visibly more compact nuclear chromatin pattern than those on non-stretched membranes (Figure 3A). Quantitative analysis showed that nuclear area remained largely unchanged, with no significant difference between groups (Figure 3B). In contrast, chromatin density was significantly increased in cells cultured on the stretched membrane, reaching approximately 1.8-fold of that in the non-stretched group (Figure 3C). These results indicate that stretch-regulated interfacial topography did not measurably alter nuclear size, but was sufficient to induce substantial chromatin reorganization in macrophages. We next asked whether this nuclear response was associated with changes in macrophage inflammatory polarization. qRT–PCR analysis showed that macrophages cultured on stretched membranes exhibited elevated expression of the pro-inflammatory genes TNF and IL-1, which increased to approximately 2.4-fold and 2.2-fold of the non-stretched group, respectively (Figure 3D). In contrast, the anti-inflammatory markers Arg1 and IL-10 showed no obvious increase and remained comparable to, or slightly below, control levels. Consistent with the transcriptional data, ELISA further demonstrated that TNF-α secretion was significantly increased in the stretched group, reaching approximately 3.8-fold of that in cells cultured on non-stretched membranes (Figure 3E). These findings support that the stretched low-roughness membrane biases RAW264.7 macrophages toward a pro-inflammatory phenotype.

To further assess whether this phenotypic shift was accompanied by functional activation, we quantified macrophage phagocytosis. RAW264.7 cells cultured on stretched membranes showed a significant enhancement in phagocytic activity, reaching approximately 2.6-fold of the non-stretched control (Figure 3F). This functional increase is consistent with the elevated inflammatory state of macrophages on the stretched surface and further supports the conclusion that stretch-regulated interfacial topography promotes pro-inflammatory response activation. Together, these results demonstrate that the stretched PDMS membrane, characterized by reduced surface roughness, induces chromatin condensation in RAW264.7 macrophages without affecting nuclear area, and simultaneously promotes pro-inflammatory polarization and phagocytic function. These findings suggest that surface roughness-regulated adhesion and cytoskeletal remodeling are coupled to nuclear reorganization and inflammatory activation in macrophages.

### 3.4 Membrane roughness regulates macrophage adhesion and chromatin accessibility

**FIGURE 4:**
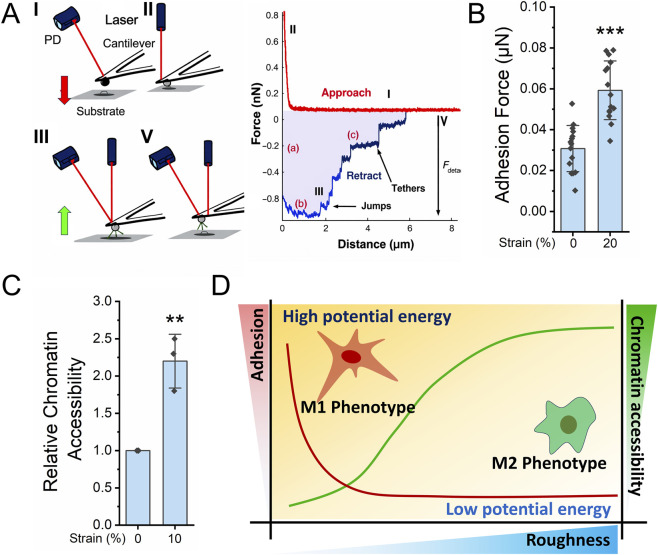
Stretch-regulated low-roughness membranes enhance macrophage adhesion and chromatin accessibility and support a roughness-dependent phenotypic model. **(A)** Schematic illustration of single-cell force spectroscopy (SCFS) used to measure macrophage adhesion to the substrate. A single RAW264.7 cell attached to a ConA-functionalized cantilever was brought into contact with the membrane surface and then retracted to record cell–substrate detachment forces. **(B)** Quantification of adhesion force between RAW264.7 macrophages and PDMS membranes in the non-stretched (0%) and stretched (20%) groups. **(C)** Quantification of relative chromatin accessibility in RAW264.7 macrophages cultured on PDMS membranes with the indicated strain levels for 12 h. **(D)** Schematic model illustrating the proposed relationship among surface roughness, adhesion strength, chromatin accessibility, and macrophage phenotype. In this model, reduced surface roughness promotes stronger adhesion and favors a pro-inflammatory state, whereas higher surface roughness is associated with weaker adhesion and a more M2-like state. Data are presented as mean ± SD. Each dot represents one independent measurement. **p < 0.01, ***p < 0.001 versus the 0% group.

To further define how stretch-regulated surface roughness controls macrophage behavior, we next quantified the physical interaction between macrophages and the PDMS membrane using single-cell force spectroscopy (SCFS). In this assay, a single macrophage attached to the cantilever was brought into contact with the membrane surface, maintained in contact for 10 s, and then retracted to measure the adhesion force between the cell and substrate (Figures 4A,B). This approach allowed direct evaluation of the strength of cell–material interaction on membranes with different roughness states.

SCFS measurements showed that macrophages on the stretched membrane exhibited significantly stronger adhesion to the substrate than those on the non-stretched membrane (Figure 4C). Quantitative analysis demonstrated that the adhesion force increased to approximately 2.1-fold of that measured on the non-stretched control. These findings are consistent with the increased FAK signal and enhanced spreading area observed in Figure 2, and further support that the low-roughness surface generated by stretching promotes stronger macrophage attachment to the material interface. Because adhesion-dependent mechanical signaling can propagate from the cell membrane to the nucleus, we next examined whether altered surface roughness also affected chromatin accessibility. Macrophages were cultured on membranes with different roughness states for 12 h, after which chromatin accessibility was quantified. As shown in Figure 4D, cells cultured on the stretched membrane displayed a significant decrease in chromatin accessibility, reaching approximately 0.63-fold of that in the non-stretched group. Together with the increased adhesion force, this result suggests that the stretch-regulated low-roughness surface not only strengthens macrophage attachment, but also reshapes the nuclear chromatin state.

Based on the above findings, we established a conceptual model linking surface roughness, cell adhesion force, chromatin accessibility, and macrophage phenotype (Figure 4). Surface roughness is proposed to regulate macrophage phenotype by coordinately modulating cell–material adhesion, intracellular mechanical state, and chromatin accessibility. On smoother surfaces, macrophages form stronger adhesive interactions with the substrate, accompanied by greater cell spreading, enhanced focal adhesion signaling, and a mechanically tenser state. These features are associated with a higher potential energy landscape that stabilizes a pro-inflammatory, phenotype. In this state, macrophages exhibit reinforced adhesion-dependent signaling and a nuclear state that is less permissive to transition away from inflammatory activation.

As surface roughness increases, stable cell–substrate adhesion is progressively reduced, leading to weaker mechanical anchorage and a shift in cytoskeletal organization. This change lowers the overall mechanical constraint imposed on the cell and moves macrophages toward a lower potential energy state. At the same time, rougher surfaces are proposed to promote a more accessible chromatin landscape, thereby facilitating transcriptional programs associated with alternative activation. Through this adhesion–nucleus coupling, macrophages gradually transition from a pro-inflammatory state on smooth surfaces to an anti-inflammatory state on rough surfaces.

The intersection of the adhesion and chromatin accessibility curves represents a transition region in which neither phenotype is fully dominant, suggesting that macrophage polarization is not switched abruptly but instead shifts progressively along the roughness gradient. Overall, this model integrates the results of Figure 4 by suggesting that surface roughness functions as a biophysical cue that reprograms macrophage fate through coordinated regulation of adhesion strength, mechanical energy state, and chromatin accessibility, ultimately biasing inflammatory polarization on smooth surfaces and reparative polarization on rough surfaces.

### 3.5 Roughened glass surfaces validate the roughness-dependent regulation of macrophage adhesion, chromatin accessibility, and inflammatory phenotype

**FIGURE 5:**
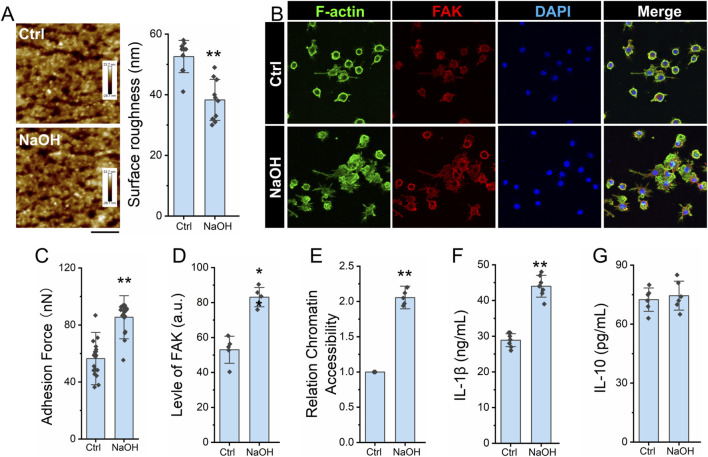
Roughness-modified glass surfaces validate the regulation of macrophage adhesion, chromatin accessibility, and inflammatory output by interfacial topography. **(A)** Representative AFM images of untreated control glass and NaOH-treated glass, together with quantification of surface roughness. **(B)** Representative immunofluorescence images of RAW264.7 macrophages cultured on control and NaOH-treated glass, followed by staining for F-actin (green), focal adhesion kinase (FAK; red), and nuclei (DAPI; blue). Merged images are shown in the right panel. Scale bar, 50 μm. **(C)** Quantification of adhesion force between RAW264.7 macrophages and glass surfaces, as measured by single-cell force spectroscopy. **(D)** Quantification of FAK fluorescence intensity in RAW264.7 macrophages cultured on control and NaOH-treated glass. **(E)** Quantification of relative chromatin accessibility in RAW264.7 macrophages cultured on the indicated glass surfaces. **(F)** Quantification of IL-1β secretion in the culture supernatant of macrophages cultured on control and NaOH-treated glass, as measured by ELISA. **(G)** Quantification of IL-10 secretion in the culture supernatant of macrophages cultured on control and NaOH-treated glass, as measured by ELISA. Data are presented as mean ± SD. Each dot represents one cell or one independent measurement. *p < 0.05, **p < 0.01 versus the control group.

To further validate the mechanistic model proposed above, we established an independent proof-of-concept system using NaOH-treated glass slides. In this experiment, glass surfaces were chemically treated with NaOH (1 mM) overnight to alter surface roughness, after which macrophages were seeded onto untreated and NaOH-treated glass for subsequent analysis of adhesion, cytoskeletal organization, chromatin accessibility, and inflammatory phenotype (Figure 5). This strategy was designed to test whether the roughness dependent macrophage response observed on stretched PDMS membranes could also be reproduced on a distinct material platform. AFM imaging showed that NaOH treatment effectively altered the surface topography of glass slides (Figure 5A). Quantitative analysis confirmed a significant enhancement of the surface roughness after treatment, which generated a distinct roughness state for biological validation. These results established the glass model as an orthogonal platform for testing the role of surface roughness in macrophage regulation.

We next examined macrophage morphology and adhesion associated signaling on these surfaces. Immunofluorescence staining showed that macrophages cultured on NaOH-treated glass exhibited a less spread morphology, accompanied by reduced FAK signal and altered F-actin organization, compared with cells on untreated control glass (Figure 5B). Consistent with these imaging results, single-cell force spectroscopy showed that macrophage adhesion force on the NaOH-treated surface decreased to approximately 1.52-fold of that on the control surface (Figure 5C). In parallel, FAK intensity decreased to approximately 1.46-fold of the control level (Figure 5D). These data indicate that roughness modulation on glass was sufficient to reduce macrophage attachment and adhesion associated signaling, consistent with the mechanosensitive behavior observed on PDMS membranes. To determine whether these interfacial changes were coupled with nuclear regulation, we further quantified chromatin accessibility. Macrophages cultured on NaOH-treated glass displayed significantly enhanced chromatin accessibility, reaching approximately 2-fold of that on untreated glass (Figure 5E). This result supports that surface roughness can regulate not only cell adhesion but also downstream nuclear chromatin state across different substrate systems.

We next assessed whether these changes were accompanied by functional inflammatory polarization. ELISA analysis showed that macrophages cultured on NaOH-treated glass exhibited a significant decrease in IL-1β secretion approximately 1.5-fold of the control group (Figure 5F), whereas IL-10 levels remained largely unchanged (Figure 5G). These findings indicate that roughness modulation on glass preferentially reduced pro-inflammatory activation rather than anti-inflammatory signaling. Taken together, these proof-of-concept results support the generalizability of our proposed model. By using an independent glass-based system, we confirmed that surface roughness is sufficient to regulate macrophage adhesion strength, focal adhesion associated signaling, chromatin accessibility, and inflammatory output. Importantly, these findings suggest that the roughness dependent macrophage response is not restricted to the stretchable PDMS platform, but may represent a broader interfacial mechanism by which material topography governs macrophage phenotype. This orthogonal validation therefore strengthens our conclusion that surface roughness acts as an upstream physical cue that organizes macrophage inflammatory behavior through coordinated regulation of adhesion and chromatin state.

## 4 Discussion

In this study, we developed a magnetic stretching device to impose controlled deformation on a thin PDMS membrane and used this platform to investigate how stretch-regulated interfacial properties influence macrophage behavior. The key finding is that membrane stretching significantly altered roughness without measurably changing the modulus of PDMS, thereby providing a useful system to separate topographical effects from substrate stiffness. Using this platform, we found that RAW264.7 macrophages cultured on the stretched membrane displayed enhanced spreading, reduced roundness, decreased F-actin intensity, increased FAK-associated signalling, increased chromatin density and decreased accessibility, elevated pro-inflammatory marker expression, increased cytokine secretion, and enhanced phagocytic activity. Together, these results support the conclusion that stretch-regulated surface roughness is sufficient to reshape macrophage inflammatory state.

A major significance of this work is that it identifies surface roughness as an active regulator of macrophage phenotype rather than a passive material feature. In biomaterial aspects, roughness changes together with stiffness, chemistry, wettability, or ligand presentation, making mechanistic interpretation difficult (Chaudhary et al., 2023; Romero-Gavilán et al., 2024). Here, AFM analysis showed that stretching smoothed the PDMS surface while leaving Young’s modulus largely unchanged, allowing us to focus on the biological consequences of interfacial topography. The morphological and molecular responses of macrophages on the stretched membrane indicate that altered roughness can strongly influence early cell–material interactions. This suggests that even modest changes in interface structure can redirect macrophage behavior and thereby shape downstream inflammatory outcomes.

Our data further supports a mechanistic link between surface roughness, adhesion, and nuclear regulation. Macrophages on the stretched membrane showed increased spreading, higher FAK signal, and stronger adhesion force by single-cell force spectroscopy, indicating more effective cell–substrate engagement on the low-roughness surface. These adhesion-related changes were accompanied by altered cytoskeletal organization and a significant increase in chromatin density and accessibility, despite no obvious change in nuclear area (Mazzocca et al., 2023; Katava et al., 2022). This pattern suggests that mechanical information generated at the material interface is transmitted through adhesion complexes and cytoskeletal remodeling to the nucleus, where it reorganizes chromatin state. Thus, the macrophage response observed here is best understood as an integrated adhesion–cytoskeleton–chromatin axis, through which physical surface cues are converted into inflammatory programming.

Compared with previous studies showing roughness mediated macrophage phenotype transitions, the present platform provides a useful approach to partially decouple surface roughness from bulk modulus. However, other interfacial variables, including ligand adsorption, wettability, and nanoscale chemistry, may still contribute and should be further quantified in future work (Li et al., 2020). In addition, macrophage responses to roughness are unlikely to follow a strict binary M1/M2 pattern. The marker profile observed here is better interpreted as a roughness-biased inflammatory activation state, and potential hybrid or intermediate phenotypes should be examined using broader transcriptomic and functional assays.

Functionally, these structural and nuclear changes were associated with a clear shift toward a pro-inflammatory phenotype (Lin et al., 2022). Macrophages cultured on the stretched membrane showed increased expression of TNF and IL-1, elevated TNF-α secretion, and enhanced phagocytic activity, whereas anti-inflammatory markers did not increase correspondingly. Importantly, this trend was further supported by the proof-of-concept experiment on NaOH-treated glass, where roughness modulation again altered macrophage adhesion, FAK signaling, chromatin accessibility, and inflammatory output. Although the precise directional relationship between roughness and phenotype should be described carefully according to the final quantified data, the overall conclusion is consistent across platforms, surface roughness governs macrophage phenotype by regulating cell adhesion strength and downstream nuclear state. This cross-platform validation strengthens the generality of the proposed model.

This work also has translational implications for the design of immune-instructive biomaterials. Macrophages are early responders to implants, scaffolds, wound materials, and local delivery systems, and their activation state critically influences integration, repair, and chronic inflammation (Na et al., 2023; Chen et al., 2023). Our findings suggest that tuning surface roughness may provide a practical way to regulate macrophage behavior through physical rather than biochemical intervention. At the same time, several limitations should be noted. The current study was performed mainly in RAW264.7 cells, and future validation in primary or human macrophages will be important. In addition, the causal molecular mediators linking adhesion to chromatin remodeling remain to be defined, and future studies should examine the roles of integrins, FAK, actomyosin tension, and nucleus-associated mechanotransduction pathways. Although the workflow was designed to reduce coating related variation, future studies using direct quantification of adsorbed ligand density or fluorescently labeled coating molecules would further strengthen this conclusion. Nevertheless, our results establish surface roughness as a mechanically tunable regulator of macrophage inflammatory phenotype and provide a framework for designing biomaterial interfaces that modulate inflammation through adhesion–chromatin coupling.

## Data Availability

The original contributions presented in the study are included in the article/supplementary material, further inquiries can be directed to the corresponding author.
